# Origin of high thermoelectric performance of FeNb_1−*x*_Zr/Hf_*x*_Sb_1−*y*_Sn_*y*_ alloys: A first-principles study

**DOI:** 10.1038/srep33120

**Published:** 2016-09-08

**Authors:** Xiwen Zhang, Yuanxu Wang, Yuli Yan, Chao Wang, Guangbiao Zhang, Zhenxiang Cheng, Fengzhu Ren, Hao Deng, Jihua Zhang

**Affiliations:** 1 Institute for Computational Materials Science, School of Physics and Electronics, Henan University, Kaifeng 475004, China; 2 Guizhou Provincial Key Laboratory of Computational Nano-Material Science, Guizhou Education University, Guiyang 550018, China

## Abstract

The previous experimental work showed that Hf- or Zr-doping has remarkably improved the thermoelectric performance of FeNbSb. Here, the first-principles method was used to explore the possible reason for such phenomenon. The substitution of *X* (Zr/Hf) atoms at Nb sites increases effective hole-pockets, total density of states near the Fermi level (*E*_*F*_), and hole mobility to largely enhance electrical conductivity. It is mainly due to the shifting the *E*_*F*_ to lower energy and the nearest Fe atoms around *X* atoms supplying more d-states to hybrid with *X* d-states at the vicinity of the *E*_*F*_. Moreover, we find that the *X* atoms indirectly affect the charge distribution around Nb atoms via their nearest Fe atoms, resulting in the reduced energy difference in the valence band edge, contributing to enhanced Seebeck coefficients. In addition, the further Bader charge analysis shows that the reason of more holes by Hf-doping than Zr in the experiment is most likely derived from Hf atoms losing less electrons and the stronger hybridization between Hf atoms and their nearest Fe atoms. Furthermore, we predict that Hf/Sn co-doping may be an effective strategy to further optimize the thermoelectric performance of half-Heusler (HH) compounds.

Thermoelectric (TE) materials can convert automobile exhaust heat and industrial waste heat into usable electrical power, which can improve today’s energy crisis and reduce green-house gas emissions. Half-Heusler (HH) compounds with the cubic structure (*F*4/3*m*) have recently gained great attention as promising thermoelectric materials due to their good mechanical strength, thermal stability, and non-toxicity. When their total valence electron count in the HH primitive cell is 18, they exhibit the non-magnetic semiconductor features with narrow energy gaps, which potentially provides a relatively large Seebeck coefficient, such as, MNiSn (M = Ti, Zr, Hf)^1^,^2^, TiCoSb^3^, and YPbSb^4^.

The thermoelectric performance is directly measured by the dimensionless figure of merit (*ZT* = *S*^2^*σT*/*κ*_*tot*_, *κ*_*tot*_ = *κ*_*latt*_ + *κ*_*e*_), here, *S, σ*, and *κ*_*tot*_ are Seebeck coefficient, electrical conductivity, and total thermal conductivity. However, HH alloys usually have a relatively low *σ* and a high *κ*_*tot*_, which results in their low thermoelectric efficiencies. In recent years, many approaches have been employed to enhance the *ZT* of the HH alloys, such as, nanocomposite approach, isoelectronic alloying, point-defects, grain size reduction, etc^5^,^6^,^7^,^8^,^9^,^10^,^11^,^12^,^13^,^14^,^15^. Joshi *et al*.^16^ reported that the *ZT* of the nanostructured p-type Nb_0.6_Ti_0.4_FeSb_0.95_Sn_0.05_ composition reached ~1 at 700 K using the cost-effective mass-production nanocomposite approach. Liu *et al*.^17^ studied that the isoelectronic substitution of Hf at the Ti sites, and the highest *ZT* = 1.1 was obtained for Zr_0.2_Hf_0.8_NiSn_0.985_Sb_0.015_ at 1000 K. Yang *et al*.^7^ predicted that alloying Pt on the Ni sites will further reduce the lattice thermal conductivity (*κ*_*latt*_) of ZrNiSn, due to the heavier mass and larger radius of Pt atoms. Bhattacharya *et al*.^18^ investigated the effect of submicron grain sizes on *κ*_*latt*_ in ball milled and shock compressed samples, and found that a reduced grain size could decrease the *κ*_*latt*_.

According to ref. 19 and the Bader charge analysis, FeNbSb can be thought as comprising an Nb^0.89+^ ion stuffing the zinc-blend (FeSb)^0.89−^ sublattice, and the valence electron count of (FeSb)^0.89−^ is 18, indicating that FeNbSb is a closed-shell and non-magnetic semiconductor. However, for FeNbSb, the relatively low power factor (*S*^*2*^*σ*) due to a low carrier concentration, and a high *κ*_*tot*_ due to a weak phonon scattering, suggest that it is not a promising thermoelectric material. Recently, Fu *et al*.^20^,^21^ reported that the substitution of *X* (Ti/Zr/Hf) atoms at Nb sites can simultaneously enhance *PF (S*^*2*^*σ*) and reduce *κ*_*latt*_. Their experimental results showed that the *ZT* values of Ti/Zr-doped FeNbSb are smaller than that of Hf-doped FeNbSb, and the *ZT* of 14% Hf-doped FeNbSb can reach 1.5 at 1200 K. Then, Fang *et al*.^22^ calculated the formation energies of Ti-/Zr-/Hf-doping with 25% doping level to explain why Hf more efficiently supplying carriers than Ti or Zr in experiment. However, the wrong formula in ref. 22 was used, and the doping level of Ti/Zr/Hf in ref. 22 is 25% which is far away from the experimental one (below 14%). Thus, their results are difficult to be used to explain the experiment result. Herein, we substitute Nb sites with Ti/Zr/Hf atoms with various doping levels, and find that Hf-doping can obviously increase effective valence band valleys (effective hole-pockets) and the total density of states (DOS) near the Fermi level (*E*_*F*_) without obviously decreasing the degeneracy of the valence band valleys, and thus, the *PF (S*^*2*^*σ*) is improved. Our work also shows that Hf-doping results in a higher hole mobility than Zr-doping by affecting on the valence band effective mass (m*) near the *E*_*F*_, and the nearest Fe atoms around Hf atoms supplying more d-states to hybrid with Hf d-states at the vicinity of the *E*_*F*_ leads to the increased effective hole-pockets. Moreover, from the further Bader charge analysis, we find that the stronger hybridization between Hf atoms and their nearest Fe atoms near the *E*_*F*_ and the less lost electrons of Hf atoms possibly result in 15.625% Hf-doped FeNbSb supplying more holes than 15.625% Zr-doped FeNbSb. In addition, the charges are localized around Nb atoms near the Zr (or Hf) atoms in FeNb_1−*x*_Zr/Hf_*x*_Sb (*x* = 0.10 and 0.15) systems, which likely leads to the reduced energy difference (⊿E_A-M_) in the valence band edge between the point A and M.

Besides, to further optimize the thermoelectric performance, we attempt the substitution of Sn atoms at Sb sites of FeNb_1−*x*_Hf_*x*_Sb (*x* = 0.15 and 0.15625) alloys. We find that 5% Sn-doping can converge the valence band valleys near the *E*_*F*_ (at the point M) and thus, obviously enhance the total Seebeck coefficient (*S*_*tot*_). The *ZT* of 5% Sn-doped FeNb_0.85_Hf_0.15_Sb alloy can reach 1.76 at 1200 K. Hence, we predict that the thermoelectric performance of FeNbSb can be largely increased by Hf/Sn co-doping.

## Results and Discussion

### Formation energies and band gaps of FeNb_1−*x*_Zr/Hf_*x*_Sb_1−*y*_Sn_*y*_

To study the stability of the doping modes, the formation energies (*E*_*form*_) were calculated by


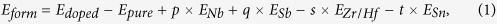


where *E*_*doped*_ is the total energy of the Zr, Hf mono-doped or Hf/Sn co-doped FeNbSb supercell and *E*_*pure*_ is the total energy of pure FeNbSb supercell. *E*_*Nb*_, *E*_*Sb*_, *E*_*Zr*_, *E*_*Hf*_, and *E*_*Sn*_ are the total energies per atom in their bulk phases, respectively. The coefficients (*p, q, s*, and *t*) represent the number of the doped or doping atoms. Before the doping sites are selected, we carried out a test that the relation between the distances among doping atoms and the formation energies of FeNb_1−*x*_Zr/Hf_*x*_Sb_1−*y*_Sn_*y*_ alloys, and find that the doping atoms tend to spread out. Thus, in the calculation of their formation energies, the selected distances among the doping atoms are all larger than (or equal to) 6 Å. Their lowest formation energies are presented in Table 1. Table 1 shows that the formation energies of Zr, Hf mono-doped systems are all negative, while Hf/Sn co-doped systems are positive due to their high Sn-doping levels. A lower doping level of Sn may induce negative formation energies. However, the lower doping level needs a larger supercell, which is difficult for us based our computation condition. In recent years, many co-doping half-Heusler systems with high doping levels have been achieved in the experiment, such as, p-type Hf_0.44_Zr_0.44_Ti_0.12_CoSb_0.8_Sn_0.2_ and p-type Nb_0.6_Ti_0.4_FeSb_0.95_Sn_0.05_, etc.^16^.

The thermoelectric transport properties of materials (such as, Seebeck coefficient and electrical conductivity) strongly depend on band structure. Hence, the accurate band gap (*E*_*g*_) is very important to estimate the thermoelectric performance. Here, we estimated the band gaps of the doping modes from the experimental data using the Goldsmid-Sharp formula:


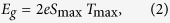


where *T*_*max*_ is the temperature at which the maximum Seebeck coefficient (*S*_*max*_) occurs^23^. The calculated band gap (0.58 eV) of FeNb_0.92_Hf_0.08_Sb is close to its estimated value (0.60 eV) from the previous experiment^19^, indicating the reasonability of the methods in our calculations.

### Structural and thermoelectric analysis of FeNbSb

**Figure 1:**
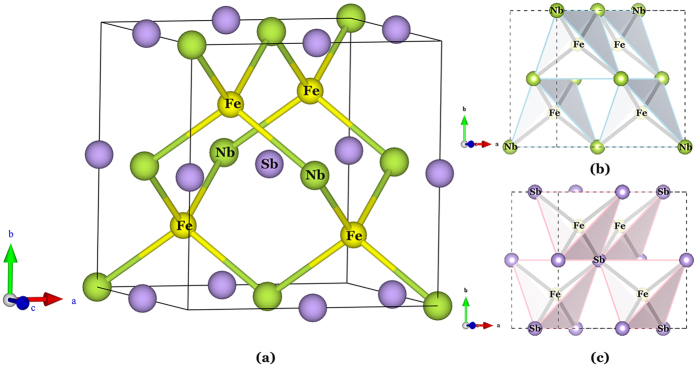
(**a**) Crystal structure of half-Heusler FeNbSb with the *F*4/3*m* space group. (**b**) Corner sharing FeNb_4_ tetrahedra. (**c**) Corner sharing FeSb_4_ tetrahedra. Color code: Fe atoms, yellow; Nb atoms, green; Sb atoms, purple.

Figure 1 shows that FeNbSb crystallizes in the cubic structure (space group: *F*4/3*m*, no. 216). Each unit cell contains four Fe atoms, four Nb atoms, and four Sb atoms. For FeNbSb, the optimized lattice constant is 5.959 Å. The system can be viewed as rock salt structure arrangement. Connecting Fe atoms and Nb atoms reveals the stuffed zinc blende lattice of the half-Heusler structure displayed in Fig. 1(a). Fe atoms are found to be set at (0.25, 0.25, 0.25), in the centers of tetrahedra formed by Nb atoms, as well as by Sb atoms as shown in Fig. 1(b,c).

The electronegativity values of Fe, Nb, and Sb are 1.83, 1.6, and 2.05, respectively. In FeNbSb, Nb atoms tend to lose electrons due to their smaller electronegativity values, while Fe and Sb atoms tend to obtain electrons due to their larger electronegativity values. To quantitative analyze the charge transfer among these atoms, we calculated the Bader charge analysis of FeNbSb using WIEN2k. The Bader charge analysis shows that each Nb atom averagely loses 0.89 |e|, and each Fe (or Sb) atom averagely obtain 0.55 |e| (or 0.34 |e|). In addition, Kandpal *et al*.^19^ showed that simple valence rules were obeyed for bonding in 8- or 18-electron half-Heusler compounds, such as, LiMgN (Li^+^(MgN)^−^) and TiCoSb (Ti^4+^(CoSb)^4−^), and thus, FeNbSb can be analogously written as Nb^0.89+^ (FeSb)^0.89−^.

The energy dependent Seebeck coefficient *S* and electrical conductivity divided by the scattering time, *σ*/*τ*, are calculated from the semiclassical transport equation as implemented in BoltzTraP code^24^. The electrons distribute in a narrow range of *μ *−* k*_*B*_*T *<* ε *<* μ* + *k*_*B*_*T* (near the chemical potential, *μ*), where *k*_*B*_ is the Boltzmann constant^25^. The transport distribution can be written as^26^


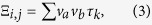


which is the kernel of all transport coefficients. Where *a* and *b* are the tensor indices, *v*_*a*_ and *v*_*b*_ are the group velocities, and *τ*_*k*_ is the relaxation time. From the rigid band approach, the electrical conductivity and Seebeck coefficient can be expressed as a function of temperature (*T*) and chemical potential (*μ*) by integrating^27^


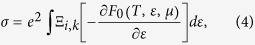



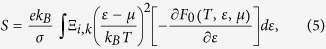


here, *F*_*0*_ is a Fermi-Dirac distribution function. The thermoelectric figures of merit (*ZT*) of TE materials are in close relation with their electronic structures. The Seebeck coefficient and electrical conductivity are both sensitive to the electronic structure at the vicinity of the *E*_*F*_. Thus, they can be increased by tuning the DOS near the edge of *E*_*F*_ by doping. Boltzmann transport theory provides a general understanding of the Seebeck coefficient that is expressed as Equation (6) and the maximum attainable figure of merit (*Z*_*max*_) of Equation (7).


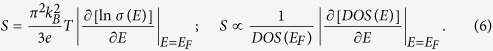


The electronic conductivity *σ (E*) is defined as a function of the band filling or Fermi energy, *E*_*F*_^28^. If electronic scattering is independent of energy, then *σ (E*) is just proportional to the density of states (DOS) at *E*.


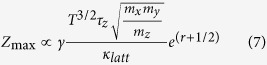


where *γ* is the degeneracy of band extrema, *τ*_*z*_ is the relaxation time of the carriers (holes or electrons) moving along the transport (*z*) direction, *r* is the scattering parameter, and *κ*_*latt*_ is the lattice thermal conductivity, which weakly depends on the electronic structure.

**Figure 2:**
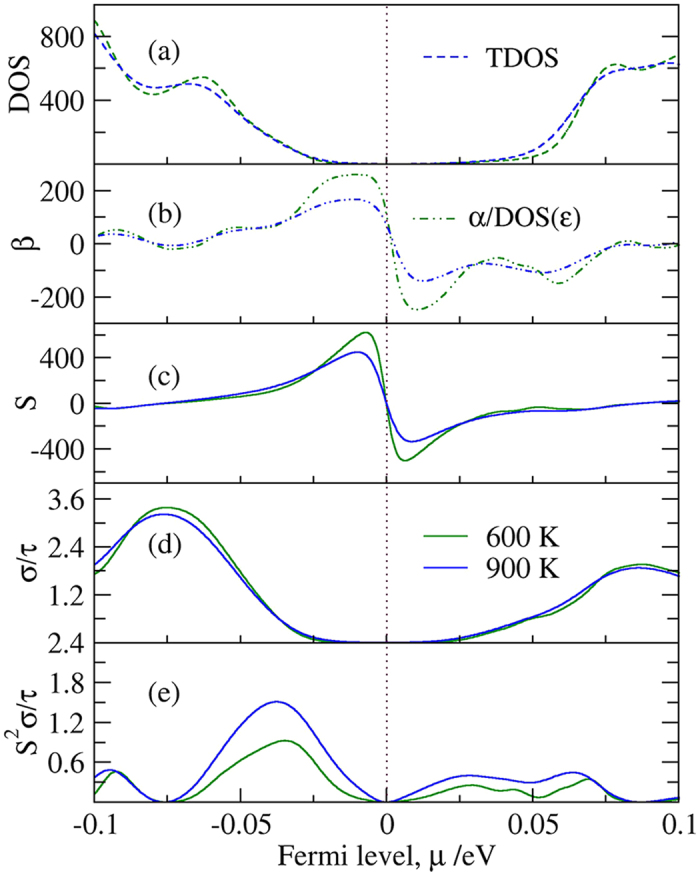
(**a**) Density of states, (**b**) the energy dependence of *β*, and (**c–e**) electrical transport properties versus the Fermi levels for pure FeNbSb. The units for TDOS, *S, σ*/*τ*, and *S*^2^*σ*/*τ* are states eV^−1^u.c.^−1^, μV^−1^K^−1^, 10^20^ Ω^−1^m^−1^s^−1^, 10^12^ WK^−2^m^−1^s^−1^, respectively. In the DOS panel of FeNbSb, the energy dependence of *α* = −*∂*[*DOS*(*ε*)]/*∂ε, β* = *α*/*DOS*(*ε*) are illustrated.

We used the rigid band approximation (RBA)^24^,^26^ to evaluate the thermoelectric transport properties of FeNbSb. In RBA, doping in FeNbSb is assumed without changing the band structure of the compound but to shift the Fermi level up or down. This is a reasonably good approximation if the doping level is not very high, and it has been widely used for theoretical study of thermoelectric materials^29^,^30^,^31^. Figure 2 plots the total DOS, energy dependence of *β (α* = −∂[*DOS*(*ε*)]/*∂ε, β* = α/*DOS*(*ε*)), and the calculated electrical transport properties versus the Fermi levels for FeNbSb at 600 K and 900 K. Although the Seebeck coefficient of half-Heusler FeNbSb has a large value when the Fermi level is close to 0 eV, and the power factor is usually small owing to low carrier concentration, and therefore, low electrical conductivity. With the Fermi level shifting upward into the conduction band (CB) or downward into the valence band (VB), the carriers (electrons or holes) increase exponentially, leading to the dramatical increase of electrical conductivity and the relatively slow reduction in Seebeck coefficient. Figure 2(a,d) show that the electrical conductivity relative to relaxation time and the total DOS have a similar trend with the shifting of the Fermi level. In fact, if electronic scattering is independent of energy, *σ*(*E*) is just proportional to the total DOS at *E*. In addition, the similar trend of the Seebeck coefficient and the energy dependence of *β* versus the Fermi levels are also observed in Fig. 2(b,c), which is consistent with Equation (6). Figure 2(e) suggests that the power factors of p-type (or n-type) doping FeNbSb at different temperatures reach their maximums at a certain Fermi level near the band edge. At 900 K, the power factors of p-type and n-type doping FeNbSb reach 1.51τ × 10^12^ WK^−2^m^−1^ and 4.46τ × 10^11^ WK^−2^m^−1^, respectively, indicating that doping is an effective way to improve the thermoelectric properties of FeNbSb.

**Figure 3:**
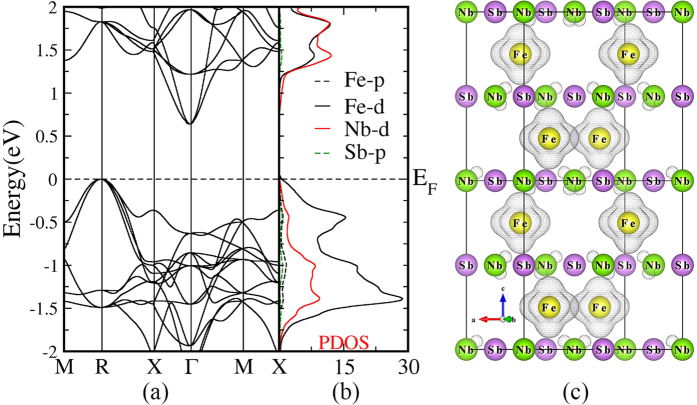
(**a**) Band structure of FeNbSb, (**b**) projected partial density of states of FeNbSb, and (**c**) calculated band decomposed charge density of FeNbSb for valence bands from −0.2 eV to the Fermi level, with the isosurface value of 0.0013. Color code: Fe atoms, yellow; Nb atoms, green; Sb atoms, purple. The Fermi level is at zero.

**Figure 4:**
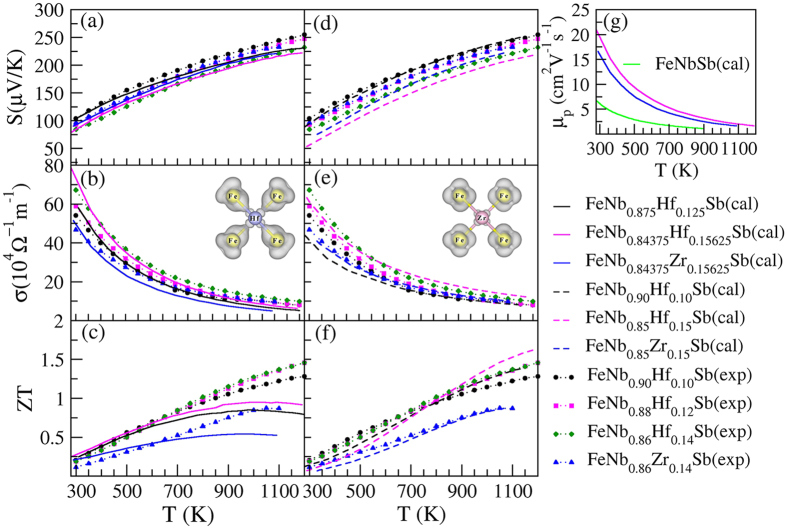
Transport coefficients of Zr, Hf mono-doped FeNbSb systems in the previous experimental work^20^ and in theoretical work as a function of temperature. The theoretical parts of doping in 2 × 2 × 2 FeNbSb supercell (**a–c**), while the theoretical parts of doping in 1 × 1 × 5 FeNbSb supercell (**d–f**). The calculated hole mobilities (*μ*_*p*_) of FeNbSb and 15.625% X-doped (Zr/Hf) FeNbSb (**g**).

The electronic structure is vital for investigating the thermoelectric transport properties. Figure 3(a,b) plot the band structures and the partial DOS from −2 eV to 2 eV. The previous reports revealed that FeNbSb are semiconductors with an indirect band gap of ~0.54 eV^21^. Our calculated indirect band gap (~0.64 eV) between the Г-R points of FeNbSb is slightly larger than 0.54 eV, which is due to the improvement in band gap by using the modified Becke Johnson (mBJ) potential. As shown in Fig. 3(a), the top of the valence band degenerates into eight sub-bands at the R point, and the bottom of the conduction band degenerates into three sub-bands at the Г point. Moreover, the dispersion of valence bands is larger than that of conduction bands. Hence, we assume that p-type FeNbSb may have a higher *Z* than n-type FeNbSb according to Equation (7). Here, we focus on investigating the p-type doping FeNbSb, and its thermoelectric transport is sensitive to the electronic states near the valence band maximum (VBM). Therefore, to deeply study the transport, the partial DOS and the partial charge densities near its VBM are presented in Fig. 3(b,c). There is little charge density around Sb atoms, and a great quantity of charge density around Fe or Nb atoms near its VBM. Thus, doping at the Sb sites to increase the hole (or electron) concentration can not obviously alter the band shape near the VBM, while doping on the Fe (or Nb) sites can effectively adjust the band structure and the DOS near the VBM. Besides, the figure of merit (*ZT*) inversely depends on the thermal conductivity (*κ*_*tot*_). Therefore, the optimal dopants not only can sharply enhance the power factor, but also can strongly reduce the thermal conductivity. Previous experimental work has shown that *X* (Ti/Zr/Hf) substitutions at Nb sites in FeNbSb can strongly create point-defect scattering of phonons to suppress lattice thermal conductivity (*κ*_*latt*_), which is due to mass fluctuation and strain field fluctuation between the host atoms and doping atoms, and simultaneously, it can effectively optimize the *PF (S*^*2*^*σ*)^20^,^21^. To deeply analyze the reason of increasing of their power factors by doping Zr/Hf atoms, we calculated the electronic structures and the transport properties of FeNb_1−*x*_Zr/Hf_*x*_Sb alloys, and then made a comparison of the calculated transport coefficients with the experimental data in Fig. 4.

### Optimization of thermoelectric performance by Zr/Hf substitutions at the Nb sites

Previous experimental and theoretical works showed that doping in the half-Heusler compounds is an effective strategy to optimize *ZT*^32^,^33^,^34^. Generally, as shown in Fig. 2, doping will decrease the Seebeck coefficient and increase the electrical conductivity. However, the optimal doping levels can achieve the balance between *S* and *σ* to improve the *PF (S*^*2*^*σ*). Fu *et al*.^20^ showed that the substitution of Zr/Hf atoms at Nb sites can obviously enhance the *PF (S*^*2*^*σ*). To clearly understand the influence of Zr/Hf atoms at Nb sites on their nearest atoms (Fe or Sb), we calculated the Bader charge analysis of 15.625% Zr- and Hf-doped FeNbSb using WIEN2k. The Bader charge analysis shows that the total electrons transferring from Zr and Hf atoms to other atoms are 7.09 |e| and 7.03 |e|, respectively. Here, Hf atoms lose less electrons, resulting in a higher hole concentration of 15.625% Hf-doped FeNbSb, which is one possible reason that Hf dopant is more efficient in supplying holes than Zr in the experiment. Moreover, our calculation showed that each nearest Fe atom of Zr (or Hf) atom averagely obtained 0.37 |e| (or 0.54 |e|) from Zr (or Hf) atom, respectively, indicating the stronger interaction of Hf-Fe than that of Zr-Fe. In fact, the stronger hybridization between Fe and Hf atoms will more obviously increase DOS, which is another possible reason that Hf dopant is more efficient in supplying holes than Zr in the experiment^20^. Furthermore, the bond lengths of Zr-Fe and Hf-Fe are 2.66 Å and 2.64 Å, respectively, also suggesting a stronger interaction between Fe atoms and Hf atoms.

Previous experimental work showed that the carrier concentration (*n*) of FeNb_1−*x*_Zr/Hf_*x*_Sb alloys changed little with the increasing of temperature. Thus, we studied the transport properties of FeNb_1−*x*_Zr/Hf_*x*_Sb as a function of temperature at a certain carrier concentration from the experimental data^20^. The electronic relaxation time (*τ*) is a necessary parameter for accurately estimating the electrical conductivity. *τ* decreases as temperature (*T*) increases at the whole temperature range, which shows a *T*^−1^ dependence. For the doping dependence, there is a standard electron-phonon interaction relaxation time, *τ* ∝ *n*^−1/3^. This yields *τ* = *C*_0_*T*^−1^*n*^−1/3^ with *τ* in s, *T* in K, and *n* in cm^−3 ^35. Moreover, for a certain carrier concentration, *n* is a constant, and thus yields *τ* = *C*_1_*T*^−1^. The calculated constants *C*_1_ for different doping levels from the experimental values were listed in Table 1. In the experiment, the Hall carrier concentration (*n*_*H*_) was calculated *via*:


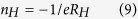



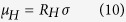


where *R*_*H*_ and *μ*_*H*_ are the Hall coefficient and the Hall mobility, respectively. In general, the transport properties of materials with small band gaps are affected by both types of carriers (electrons and holes)^36^. The weight Hall coefficient (*R*_*H*_) can be defined as:


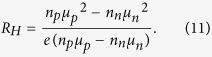


Here, *n*_*p*_ and *n*_*n*_ are the hole and electron concentration, respectively, and *μ*_*p*_ and *μ*_*n*_ are the hole and electron mobility. For p-type doping, the holes have much higher weight mobility than the electrons. However, in the theoretical calculation, because a single carrier type is assumed in Equation (9), the calculated carrier concentration (*n*_*p*_ or *n*_*n*_) is slightly smaller than the measured one. It may be an unneglectable reason of the deviation between our calculation of the transport properties and previous experimental data.

Temperature dependence of the calculated and previous experimental transport coefficients of FeNb_1−*x*_Zr/Hf_*x*_Sb alloys are shown in Fig. 4. Two supercell models were used to calculate the thermoelectric properties of FeNb_1−*x*_Zr/Hf_*x*_Sb alloys owing to little difference in their formation energies. Here, we calculated the transport properties of FeNb_1−*x*_Zr/Hf_*x*_Sb (*x* = 0.125 and 0.15625) alloys in 2 × 2 × 2 FeNbSb supercell and FeNb_1−*x*_Zr/Hf_*x*_Sb (*x* = 0.10 and 0.15) alloys in 1 × 1 × 5 FeNbSb supercell (Fig. 4). The calculated transport coefficients (*S* and *σ*) of FeNb_1−*x*_Zr/Hf_*x*_Sb (*x* = 0.125 and 0.15625) alloys agree well with the previous experimental data from 300 K to 500 K, while they are obviously lower than those in the experiment from 500 K to 1200 K, which leads to the lower *ZT* values in the theoretical work at relatively high temperatures. However, the calculated transport coefficients of FeNb_1−*x*_Zr/Hf_*x*_Sb (*x* = 0.10 and 0.15) alloys are mostly consistent with the experimental results in the whole temperature range. In addition, we find that their Seebeck coefficients gradually decrease and electrical conductivities dramatically increase with the increasing of doping levels at the studied doping range and a high content Hf can more effectively improve *PF (S*^2^*σ*) than Zr.

**Figure 5:**
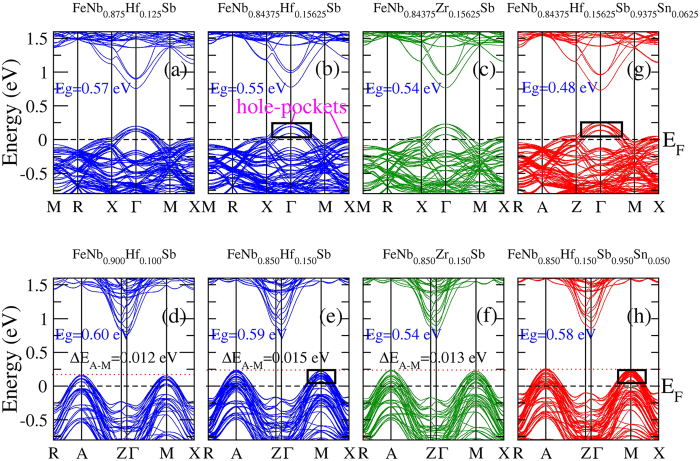
The calculated band structures of Zr, Hf mono-doped and Hf/Sn co-doped FeNbSb systems using 2 × 2 × 2 (**a–c,g**) and 1 × 1 × 5 (**d–f,h**) FeNbSb supercell. The valence band valleys (hole-pockets) at the vicinity of the *E*_*F*_ play a major role on their transport properties.

**Figure 6:**
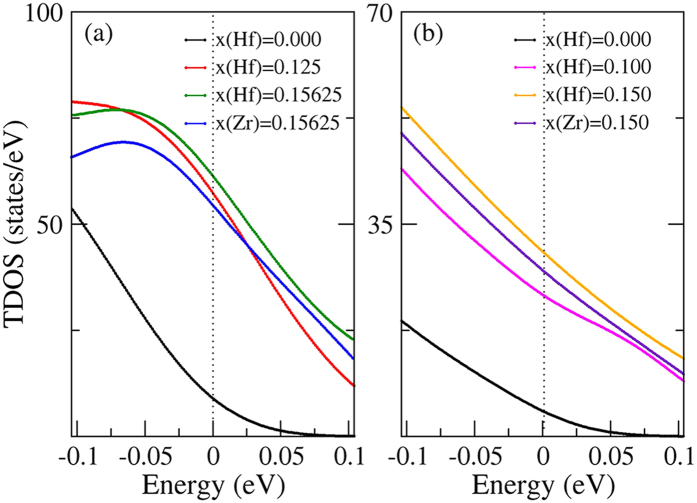
Calculated total density of states of Zr, Hf mono-doped FeNbSb alloys in 2 × 2 × 2 (**a**) and 1 × 1 × 5 (**b**) FeNbSb supercell from −0.104 eV to 0.104 eV.

The electronic structures of FeNb_1−*x*_Zr/Hf_*x*_Sb alloys were calculated to analyze their different transport coefficients using the first-principles method (Figs 5 and 6). It is known that multiple band valleys (multiple carrier-pockets) and large valley degeneracy are beneficial to achieve high *ZT*^37^,^38^,^39^. Band degeneracy increases when multiple bands have the same or comparable energy within *k*_*B*_*T*, which can contribute to increasing the Seebeck coefficient (*S*_*tot*_). If the number of the valence band valleys (or conduction band valleys) near the Fermi level (*E*_*F*_) is *m* in the FeNb_1−*x*_Zr/Hf_*x*_Sb systems, the total electrical conductivity (*σ*_*tot*_) and Seebeck coefficient (*S*_*tot*_) can be expressed as:


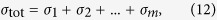



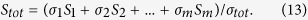


Here, the subscripts, *m (m* = 1, 2,…), refer to the transport properties of carriers in individual band. The total Seebeck coefficient is a weighted average of the Seebeck coefficients of the individual bands, and the band with the higher conductivity is more strongly weighted. The total Seebeck coefficient will be closer to the smaller Seebeck coefficient of the individual bands owing to *S* and σ having a reversely dependence of carrier concentration (*n*), and only when these individual bands are degenerate, then the *S*_*tot*_ can be maintained. In other words, the convergence of the individual bands contributes to a high *S*_*tot*_. In fact, the band valleys near the *E*_*F*_ can be called “carrier-pockets” with holding many carriers, which will induce a high electrical conductivity, originating from the increased total DOS near the *E*_*F*_. (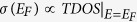) Band engineering (carrier-pocket engineering) in a bulk material is an effective strategy to improve the *PF (S*^*2*^*σ*), because it can simultaneously enhance *S*_*tot*_ and *σ*_*tot*_.

As we know, the transport coefficients are sensitive to the band structures and total DOS in a narrow range of *E*_*F*_−*k*_*B*_*T < ε < E*_*F*_ + *k*_*B*_*T (k*_*B*_*T* = 0.104 eV, *T* = 1200 K). To clearly analyze the different transport coefficients of FeNb_1−*x*_Zr/Hf_*x*_Sb alloys, we calculated their band structures and total DOS from −0.104 eV to 0.104 eV, as shown in Figs 5 and 6. The valence band valleys (or hole-pockets) in the energy range from −0.104 eV to 0.104 eV will effectively affect the thermoelectric performance, which are called “effective valence band valleys” or “effective hole-pockets”. The comparison of electronic structures of FeNb_1−*x*_Zr/Hf_*x*_Sb with FeNbSb shows that the substitution of Zr/Hf atoms at Nb sites increases the effective valence band valleys (effective hole-pockets) and the total DOS near the *E*_*F*_ except reducing the band gaps, which likely leads to a sharp increase in their *σ*_*tot*_. With shifting the *E*_*F*_ down, not only the valence band valleys at point Г but also the valence band valleys at point X become effective valence band valleys, which is a possible reason of the increased effective hole-pockets and total DOS. As for other possible reasons, I will further study later. In fact, according to Equation (12) and Equation (13), a sharp increase in the *σ*_*tot*_ will result in the dramatically decreased *S*_*tot*_. Here, we find that the valence band valleys (hole-pockets) and the total DOS near the *E*_*F*_ increase with the increasing of Hf content, and a high Hf content can more effectively increase the number of effective hole-pockets than Zr without obviously decreasing the valley degeneracy, and thus will largely improve *PF (S*^*2*^*σ*). In addition, the larger mass of Hf atom and radius difference between the host atom and Hf atom than Zr atom can effectively reduce *κ*_*latt*_, indicating that Hf atoms are good dopants^20^.

Previous experimental results and our present calculations show that a high Hf content can more effectively enhance *ZT* value than Zr. To clearly understand the reason, we plot the band structures and total DOS of FeNb_1−*x*_Hf_*x*_Sb (*x* = 0.15 and 0.15625) and FeNb_1−*x*_Zr_*x*_Sb (*x* = 0.15 and 0.15625). At the same doping level, Hf-doped FeNbSb have more effective valence band valleys (effective hole-pockets) and larger total DOS near the *E*_*F*_ than Zr-doped FeNbSb, which is the possible reason that the former has a higher *σ*_*tot*_ than the latter. Moreover, from Equation (13), we can see that the high *σ*_*tot*_ will further decrease the *S*_*tot*_, which may be a major reason that Hf-doped FeNbSb has a smaller *S*_*tot*_ than Zr-doped FeNbSb at a same doping level.

The carrier mobility (*μ*) is related to carrier concentration (*n*) and the electrical conductivity (*σ*), *μ* = *σ*/*ne* with *σ* in Ω^−1^cm^−1^, *n* in cm^−3^, and *e* in C. Then, the hole mobilities (*μ*_*p*_) of FeNbSb and 15.625% *X*-doped (Zr, Hf) FeNbSb are calculated, and their results are shown in Fig. 4(g). Figure 4(g) shows that the *μ*_*p*_ of FeNbSb is obviously increased by Zr/Hf-doping, and at the same doping level, Hf-doped FeNbSb has a higher *μ*_*p*_ than Zr-doped FeNbSb. In fact, the *μ* is proportional to the relaxation times (*τ*) and inversely proportional to effective mass (m*), *μ* = *τe*/m* 40. The calculated m* and *τ* at the different temperature are shown in supplemental Table S2. The m* decreases and *τ* increases by Zr/Hf-doping, and at the same doping level, Zr-doped FeNbSb has a larger m* than that of Hf-doped FeNbSb, while their *τ* have little difference. It indicates that the doping by Zr or Hf increase *μ*_*p*_ by directly affecting on the m* and *τ*, and the different dopant type of Zr and Hf has a stronger effect on the m* than *τ*.

**Figure 7:**
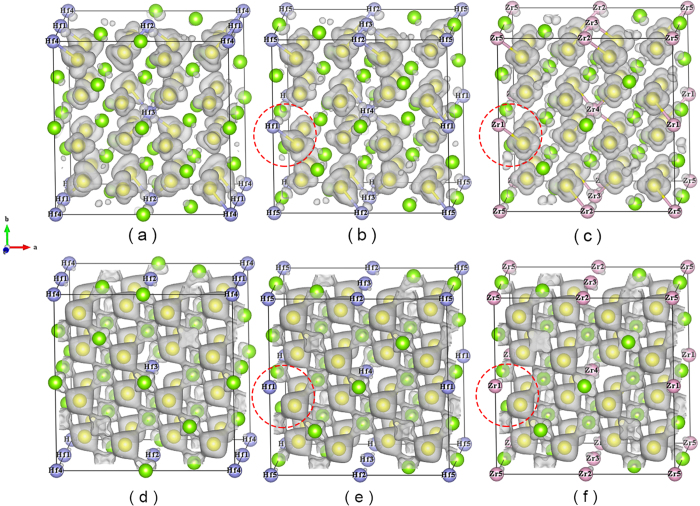
Calculated partial charge densities of FeNb_0.875_Hf_0.125_Sb (**a,d**), FeNb_0.84375_Hf_0.15625_Sb (**b,e**), and FeNb_0.84375_Zr_0.15625_Sb (**c,f**) for valence bands; (**a–c**) from −0.104 eV to 0.104 eV, isosurface value 0.0008; (**d–f**) from −2.104 eV to −0.104 eV, isosurface value 0.043. Color code: Fe atoms, yellow; Nb atoms, green; Sb atoms, purple; Hf atoms, light blue; Zr atoms, pink. Here, Sb atoms are not displayed.

**Figure 8:**
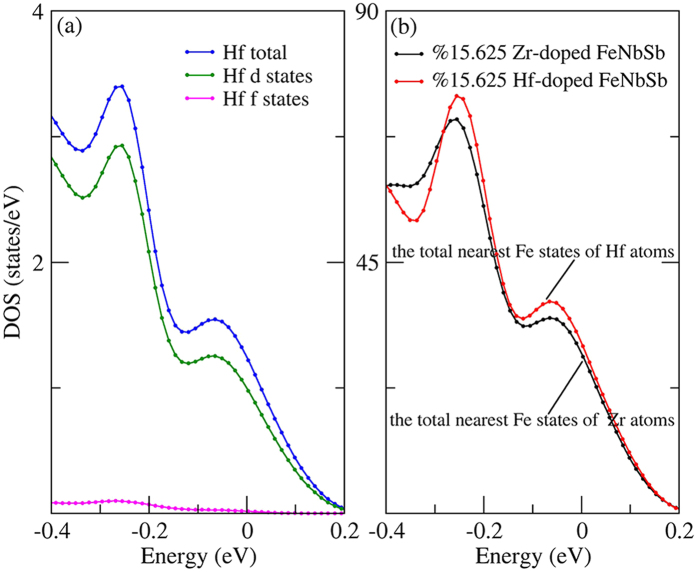
Calculated total and partial density of states of Hf atoms for 15.625% Hf-doped FeNbSb (**a**) and the total nearest Fe states of *X* (Zr/Hf) atoms for 15.625% *X*-doped FeNbSb (**b**).

In addition, to deeply study the influence of different dopants (Zr/Hf) and different doping contents, we calculated the partial charge densities of FeNb_1−*x*_Zr/Hf_*x*_Sb (*x* = 0.125 and 0.15625) alloys for valence bands from −0.104 eV to 0.104 eV and for valence bands from −2.104 eV to −0.104 eV. From Figs 3 and 7, we find that Hf atoms obviously change the charge distribution around Fe and Nb atoms, while Zr atoms have a relatively weak effect on their charge distribution, and additionally, the doping atoms (Zr/Hf) have more obvious hybridization with Fe atoms than Nb atoms near the *E*_*F*_. Figure 7(a–c) show that Zr/Hf atoms have a much stronger hybridization with their nearest Fe atoms than Nb atoms from −0.104 eV to 0.104 eV, while, Fig. 7(d–f) show that the hybridizations between Zr/Hf atoms and Fe atoms become much weaker than that between Nb and Fe atoms from −2.104 eV to −0.104 eV, indicating that the strong hybridization between Zr/Hf atoms and Fe atoms only occurs in the vicinity of *E*_*F*_. It suggests that the increased effective hole-pockets and total DOS of *X*-doped FeNbSb, and decreased m* are partially derived from the stronger hybridization between *X* d-states and their nearest Fe d-states near the *E*_*F*_. Near the *E*_*F*_, the contribution of Hf f-states can be ignored, and Hf atoms make their nearest Fe atoms supply more states to participate in transport than Zr atoms (Fig. 8). It can well explain why Hf-doped FeNbSb has more effective hole-pockets than Zr-doped FeNbSb. Additionally, the *σ*_*tot*_ of 15.625% Hf-doped FeNbSb is higher than that of 12.5% Hf-doped FeNbSb, which is likely derived from the fact that a higher Hf content can make their nearest Fe atoms supply more d-states to hybrid with Hf d-states and to participate in transport.

**Figure 9:**
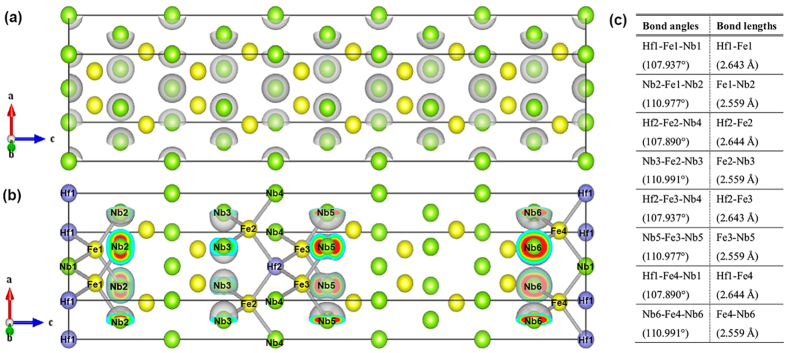
Calculated band decomposed charge densities of 1 × 1 × 5 FeNbSb supercell (**a**) and FeNb_0.9_Hf_0.1_Sb (**b**) for the valence bands at the point M, with the isosurface value of 0.01. Color code: Fe atoms, yellow; Nb atoms, green; Sb atoms, purple; Hf atoms, light blue. Here, Sb atoms are not displayed.

Besides, from Figs 3(a) and 5(d–f), we find that Zr or Hf-doping decreases the energy difference (⊿E_A-M_) in the valence band edge between the point A and M, and their calculated values are: for FeNbSb supercell, ⊿E_A-M_ = 0.040 eV; for 10% Hf-doped FeNbSb, ⊿E_A-M_ = 0.012 eV; for 15% Hf-doped FeNbSb, ⊿E_A-M_ = 0.015 eV; for 15% Zr-doped FeNbSb, ⊿E_A-M_ = 0.013 eV. Many previous studies showed that the bands were effectively degenerate when multiple bands have the same or comparable energy within *k*_*B*_*T*. Near the *E*_F_, the reduced ⊿E_A-M_ makes the band degeneracy increase, and thus, results in the enhanced *S*_*tot*_. To investigate the reason of the decreased energy difference (⊿E_A-M_) in the valence band edge, we plot the calculated band decomposed charge density of 1 × 1 × 5 FeNbSb supercell and FeNb_0.9_Hf_0.1_Sb for valence bands at the high symmetry point M in Fig. 8. Compared with FeNbSb supercell, the charge distribution of FeNb_0.9_Hf_0.1_Sb is highly localized, and these charges mainly distribute around Nb2, Nb3, Nb5, and Nb6 atoms at the vicinity of Hf atoms. In fact, Fe1 (or Fe4) atoms are located in the centers of tetrahedra formed by Hf1 and Nb2 (or Nb6) atoms, and also, Fe2 (or Fe3) atoms are located in the centers of tetrahedra formed by Hf2 and Nb3 (or Nb5) atoms, suggesting that Hf atoms indirectly affect the charge distribution around Nb atoms via their nearest Fe atoms. To deeply understand the influence of Hf-doping on the charge distribution, we calculated the bond lengths and bond angles of the tetrahedra formed by Hf and Nb atoms in Fig. 9(c). From Fig. 9(c), we find that the bond lengths and bond angles of 1-tetrahedra are same as those of 2-tetrahedra, and also, 3-tetrahedra and 4-tetrahedra have the same bond lengths and bond angles. (1, 2, 3, and 4 represent Hf1-Nb1-Nb2-Nb2, Hf2-Nb3-Nb3-Nb4, Hf2-Nb4-Nb5-Nb5, and Hf1-Nb1-Nb6-Nb6, respectively). It likely leads to the similar charge distribution around Nb2 and Nb3 atoms, and also, the similar charge distribution around Nb5 and Nb6 atoms. The obvious change in charge distribution by Hf-doping will affect the energy of the valence bands at point M, and therefore, it results in the decreased energy difference in the valence band edge between the point A and M.

### Further optimization of thermoelectric performance by Sn substitutions at the Sb sites

Many previous experimental works showed that the substitution of Sn atoms at Sb sites or the co-doping (*X*/Sn) strategy could effectively improve the thermoelectric performance of the HH compounds^16^,^41^,^42^. To further improve the thermoelectric performance of Hf-doped FeNbSb alloys, we attempt to substitute Sb sites by Sn atoms in FeNb_1−*x*_Hf_*x*_Sb (*x* = 0.15 and 0.15625) alloys. The calculated results show that the substitution of Sn atoms at Sb sites in FeNb_1−*x*_Hf_*x*_Sb (*x* = 0.15 and 0.15625) alloys can effectively enhance *ZT,* which stimulates our interest in further investigating the Hf/Sn co-doped FeNbSb. Here, for the large doping systems, the calculation of their thermal conductivities is difficult, and thus, the experimental thermal conductivity of FeNb_0.86_Hf_0.14_Sb was used to roughly estimate the *ZT* vlaues of Hf/Sn co-doped FeNbSb systems.

**Figure 10:**
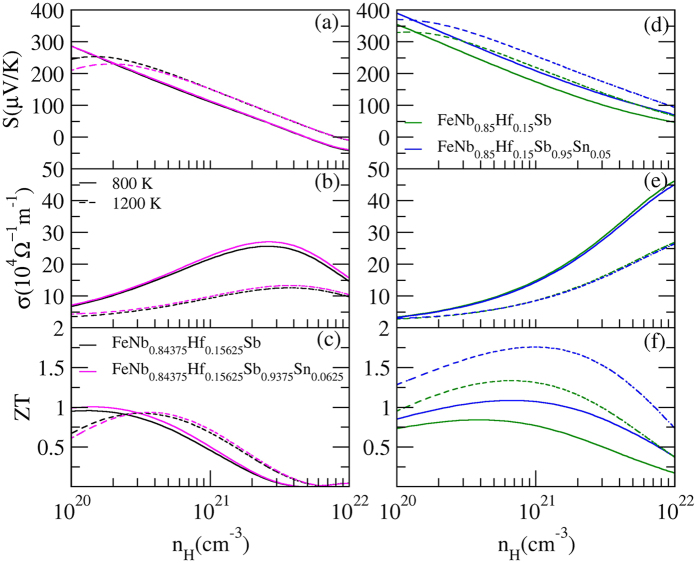
Comparison of the transport properties of Hf/Sn co-doped FeNbSb alloys with Hf mono-doped ones as a function of hole concentration from 1020 to 10^22^ cm^−3^ at 800 K and 1200 K.

As shown in Fig. 10(a–c), 6.25% Sn-doping increases the electrical conductivities without decreasing the Seebeck coefficients, leading to their enhanced *ZT*. Form Fig. 10(d–f), we can see that 5% Sn-doping simultaneously increases the electrical conductivities and Seebeck coefficients, and thus largely improve *ZT*. To clearly understand their thermoelectric transports, we plot the calculated band structures of Hf/Sn co-doped FeNbSb systems in Fig. 5(g,h). We can find that the 6.25% Sn-doping can make the valence band valleys weakly converge without decreasing the number of valence band valleys near the *E*_*F*_ and make the band gap slightly decrease, which results in little change in the Seebeck coefficients and slightly increasing of the electrical conductivities (Fig. 5(b,g)). However, Fig. 5(e,h) show that 5% Sn-doping can make the valence band valleys rather strongly converge near the *E*_*F*_ (at point M), leading to the effective increase of Seebeck coefficients. Besides, Hf/Sn co-doping will create more point-defects to further suppress *κ*_*latt*_, being beneficial to enhancing *ZT*. Hence, the thermoelectric performance of FeNb_1−*x*_Hf_*x*_Sb (*x* = 0.15 and 0.15625) alloys can be effectively improved by the substitution of Sn atoms at Sb sites.

## Conclusions

The electronic structures and thermoelectric transport properties of the Zr, Hf mono-doped and Hf/Sn co-doped FeNbSb were studied by the first-principles calculations. Our calculation shows that a high Hf content can more effectively increase the effective hole-pockets, total DOS near the *E*_*F*_, and hole mobility without obviously decreasing the valley degeneracy, likely originating from the two possible reasons. Firstly, with shifting the *E*_*F*_ down, the valance band valleys at point Г and X simultaneously make a contribution to their thermoelectric transports. Secondly, the stronger hybridization between Hf atoms and their nearest Fe atoms may decrease m* by increasing the band dispersion near the *E*_*F*_, and the Hf atoms make their nearest Fe atoms supply more d-states to participate in transport. In addition, from the further Bader analysis, we find that the less lost electrons of Hf atoms and the stronger hybridization between Hf atoms and their nearest Fe atoms most likely lead to Hf atoms more effectively supplying holes in the experiment. Their power factors (*S*^2^*σ*) are largely improved, and the average figure of merit (*ZT*) of 15% Hf-doped FeNbSb reaches ~1.62. Hence, if the formation energy increases little, the thermoelectric performance of FeNbSb may be further improved by increasing the Hf content (>14%). Furthermore, to further optimize the thermoelectric performance, the substitution of Sn atoms at Sb sites in FeNb_1−*x*_Hf_*x*_Sb (*x* = 0.15 and 0.15625) alloys was studied. The calculated results show that 5% Sn-doping can make the valence bands converge near the *E*_*F*_ (at the point M) to obtain a larger total Seebeck coefficient (*S*_*tot*_), and the *ZT* of 5% Sn-doped FeNb_0.85_Hf_0.15_Sb can reach 1.75 with the hole concentration of 1.2 × 10^21^ cm^−3^ at 1200 K. Thus, Hf/Sn co-doping may be an effective strategy for further improving the thermoelectric performance of HH compounds.

## Models and Methods

### Computational details

The full-potential linearized augmented plane wave method^43^ was used to calculate the electronic structures of FeNb_1−*x*_Zr/Hf_*x*_Sb_1−*y*_Sn_*y*_ alloys and the modified semi-local Beck-Johnson exchange potential (TB-mBJ)^44^,^45^ was used to improve band gaps, as implemented in WIEN2k^46^,^47^,^48^. The muffin-tin radii were chosen to be 2.40 a.u. for Fe and Nb, and 2.24 a.u., 2.31 a.u., 2.48 a.u., and 2.49 a.u. for Sb, Sn, Hf, and Zr, respectively. The cutoff parameter *R*_*MT*_ × *K*_*max*_ = 7 (*R*_*MT*_ is the smallest atomic sphere radius of all atomic spheres, and *K*_*max*_ is the magnitude of the largest *k* vector) was set, and the self-consistent was calculated with 100 special *k-*points in the irreducible Brillouin zone. (For FeNbSb, 1000 special *k-*points in the irreducible Brillouin zone were chosen.) Their Seebeck coefficients and electrical conductivities relative to the relaxation time were then calculated using Boltzmann semiclassical theory, as implemented in the BoltzTraP code^24^. The constant scattering time approximation based on the assumption that the scattering time determines the electrical conductivity and does not vary greatly with energy on the scale of *k*_*B*_*T* the was employed, which has been applied on many thermoelectric materials, such as Zintl phases^49^ and Cu-based compounds^50^. Here, spin-polarized density functional theory was used to test the magnetism of FeNb_1−*x*_Zr/Hf_*x*_Sb_1−*y*_Sn_*y*_ systems, and their total magnetic moments are close to zero, suggesting that they are non-magnetic and semiconductor. In addition, for heavy element Hf, relativistic effects were included.

### Structural models

In our work, two supercell models (2 × 2 × 2, 1 × 1 × 5) were adopted to simulate the different doping levels. The formation energies of doping in the 2 × 2 × 2 FeNbSb supercell were slightly lower than those of doping in the 1 × 1 × 5 FeNbSb supercell at a same doping level. However, the achieved doping levels in 1 × 1 × 5 FeNbSb supercell (10% and 15%) were much closer to those in the experiment (10% and 14%), which might be better to explain the experimental phenomena^20^. Moreover, the calculated transport properties of FeNb_1−*x*_Zr/Hf_*x*_Sb (*x* = 0.10 and 0.15) using the 1 × 1 × 5 FeNbSb supercell, agreed well with the experimental data^20^. For FeNb_1*−x*_Zr/Hf_*x*_Sb_1*−y*_Sn_*y*_ (*x* = 0.125, *y* = 0.00; *x* = 0.15625, *y* = 0.00; *x* = 0.15625, *y* = 0.0625) alloys, we constructed a 2 × 2 × 2 FeNbSb supercell, and then replaced Nb atoms with Zr/Hf atoms and substituted Sb atoms with Sn atoms in a 96-atoms cell. For FeNb_1−*x*_Zr/Hf_*x*_Sb_1−*y*_Sn_*y*_ (*x* = 0.10, *y* = 0.00; *x* = 0.15, *y* = 0.00; *x* = 0.15, *y* = 0.05) alloys, we constructed a 1 × 1 × 5 FeNbSb supercell, and then the substitution of Zr/Hf atoms at Nb sites and the substitution of Sn atoms at Sb sites was done in 60-atoms cell. To accurately understand the effect of Zr/Hf and Sn dopants on the electronic structure and transport property of FeNb_1−*x*_Zr/Hf_*x*_Sb_1−*y*_Sn_*y*_ alloys, the shortest distances among the doping atoms should be as large as possible. The most stable doping sites in FeNbSb supercell are listed in Supplemental Table S2.

## Additional Information

**How to cite this article**: Zhang, X. *et al*. Origin of high thermoelectric performance of FeNb_1−*x*_Zr/Hf_*x*_Sb_1−*y*_Sn_*y*_ alloys: A first-principles study. *Sci. Rep.* **6**, 33120; doi: 10.1038/srep33120 (2016).

## Supplementary Material

Supplementary Information

## Figures and Tables

**Table 1:** The total number of atoms (*N*) in FeNbSb supercell, total energy of doped FeNbSb (*E*_*doped*_ in eV), total energy of FeNbSb supercell (*E*_*pure*_ in eV), total energies of the individual atoms *E*_*X*_ (*X* = *Nb, Sb, Hf, Zr, Sn*) in their bulk phases (*E*_*X*_ in eV), and calculated constants (*C*_1_ in Ks) from the previous experimental data^20^.

Doping levels	*N*	*E*_*doped*_	*E*_*pure*_	*E*_*Nb*_	*E*_*Sb*_	*E*_*Hf/Zr*_	*E*_*Sn*_	*E*_*form*_	*C*_*1*_
12.5% Hf	96	−756.322	−756.934	−10.218		−9.928		−0.548	7.07 × 10^−12^
15.625% Hf	96	−756.151	−756.934	−10.218		−9.928		−0.667	6.49 × 10^−12^
15.625% Zr	96	−749.842	−756.934	−10.218		−8.521		−1.394	6.43 × 10^−12^
15.625% Hf, 6.25% Sn	96	−753.829	−756.934	−10.218	−4.146	−9.928	−3.808	+0.979	
10% Hf	60	−472.780	−473.088	−10.218		−9.928		−0.272	6.52 × 10^−12^
15% Hf	60	−472.625	−473.088	−10.218		−9.928		−0.407	5.92 × 10^−12^
15% Zr	60	−468.866	−473.088	−10.218		−8.521		−0.869	5.52 × 10^−12^
15% Hf, 5% Sn	60	−471.467	−473.088	−10.218	−4.146	−9.928	−3.808	+0.413	
