# Supplementary material for: Origin of high thermoelectric performance of FeNb1−xZr/HfxSb1−ySny alloys: A first-principles study
Source: Sci Rep. 2016 Sep 8;6:33120. doi: 10.1038/srep33120 (PMC5015081; doi:10.1038/srep33120)
Supplement: Supplementary Information [file srep33120-s1.pdf]

Supplementary Information

Origin of high thermoelectric performance of $FeNb_{1-x}Zr/Hf_xSb_{1-y}Sn_y$ alloys: A first principles study

Xiwen Zhang[1], Yuanxu Wang[1, 2,] *, Yuli Yan[1], Chao Wang[1], Guangbiao Zhang[1], Zhenxiang Cheng[1], Fengzhu Ren[1], Hao Deng[1], Jihua Zhang[1, 2]

[1]*Institute for Computational Materials Science, School of Physics and Electronics, Henan University, Kaifeng 475004, China*
[2]*Guizhou Provincial Key Laboratory of Computational Nano-Material Science, Guizhou Education University, Guiyang 550018, China*

*To whom correspondence should be addressed; e-mail: wangyx@henu.edu.cn

Table S1. The calculated effective mass (m^*) and relaxation time (τ) of FeNbSb and 15.625% *X*-doped (Zr, Hf) FeNbSb at 300 K, 600 K, and 900 K, respectively.[1,2]

Compounds	Calculated parameters	300 K	600 K	900 K
FeNbSb	m^*	-1.108 m_e	-1.103 m_e	-1.095 m_e
	τ	5.092×10^{-15}	1.877×10^{-15}	1.030×10^{-15}
15.625% Zr-doped FeNbSb	m^*	-1.0452 m_e	-1.0344 m_e	-1.0292 m_e
	τ	2.143×10^{-14}	1.072×10^{-14}	7.144×10^{-15}
15.625% Hf-doped FeNbSb	m^*	-0.9290 m_e	-0.9236 m_e	-0.9195 m_e
	τ	2.163×10^{-14}	1.082×10^{-14}	7.211×10^{-15}

Table S2. The most stable doped sites in the 2×2×2 and 1×1×5 FeNbSb supercells.

	Compound	Hf1/Zr 1	Hf2/Zr 2	Hf3/Zr 3	Hf4/Zr 4	Hf5/Zr 5	Sn1	Sn2
2×2×2 FeNbSb supercell	$Fe_{32}Nb_{28}Hf_4Sb_{32}$ 12.5%Hf	(0.0,0.0, 0.5)	(0.5,0.0, 0.5)	(0.5,0.5, 0.5)	(0.0,0.0, 0.0)			
	$Fe_{32}Nb_{27}Hf_5Sb_{32}$ 15.625%Hf	(0.0,0.5, 0.0)	(0.5,0.0, 0.0)	(0.5,0.0, 0.5)	(0.5,0.5, 0.5)	(0.0,0.0, 0.0)		
	$Fe_{32}Nb_{27}Zr_5Sb_{32}$ 15.625%Zr	(0.0,0.5, 0.0)	(0.5,0.0, 0.0)	(0.5,0.0, 0.5)	(0.5,0.5, 0.5)	(0.0,0.0, 0.0)		
	$Fe_{32}Nb_{27}Hf_5Sb_{30}Sn_2$	(0.0,0.	(0.5,0.	(0.5,0.	(0.5,0.	(0.0,0.	(0.5,0.	(0.5,0.

	15.625%Hf,6.25%Sn	5,<br>0.0)	0,<br>0.0)	0,<br>0.5)	5,<br>0.5)	0,<br>0.0)	5,<br>0.25)	5,<br>0.75)
1×1×5 FeNbSb<br>supercell	$Fe_{20}Nb_{18}Hf_2Sb_{20}$<br>10%Hf	(0.0,0.<br>0,<br>0.0)	(0.5,0.<br>5,<br>0.4)					
	$Fe_{20}Nb_{17}Hf_3Sb_{20}$<br>15%Hf	(0.0,0.<br>5,<br>0.9)	(0.5,0.<br>0,<br>0.7)	(0.5,0.<br>0,<br>0.3)				
	$Fe_{20}Nb_{17}Zr_3Sb_{20}$<br>15%Zr	(0.0,0.<br>5,<br>0.9)	(0.5,0.<br>0,<br>0.7)	(0.5,0.<br>0,<br>0.3)				
	$Fe_{20}Nb_{17}Hf_3Sb_{19}Sn_1$<br>15%Hf,5%Sn	(0.0,0.<br>5,<br>0.9)	(0.5,0.<br>0,<br>0.7)	(0.5,0.<br>0,<br>0.3)			(0.0,0.<br>0,<br>0.1)	

Effective mass calculation

If the different band effective mass near the Fermi level (E_F) is m_i^* (i= 1, 2, 3, …), and their corresponding energies are E_i (i= 1, 2, 3, …), respectively. For the doping systems, each effective mass (m_i^*) near the E_F has a Fermi weight ($f\left(E_i\right)$). Then, the m^* can be expressed as

$$m^* = \sum_{i}^{i=1} m_i^* \times f\left(E_i\right) = \sum_{i}^{i=1} m_i^* \times \frac{1}{e^{\frac{E_i - E_F^T}{kT}} + 1}, \tag{1}$$

where, E_F^T represents the Fermi levels at the different temperatures (T). Here, for such large doping systems, the calculation of all m_i^* is very difficult based the current calculation condition. In fact, in 15.625% X-doped (Zr/Hf) FeNbSb, the valence bands near the point Γ and X make a major contribution to transport, and thus for simplicity, we assume that only the m_i^* near the point Γ and X contribute to their transport.

References

1. Young, D. P., Khalifah, P., Cava, R. J. & Ramirez, A. P. Thermoelectric properties of pure and doped FeMSb (M=V, Nb). *J. Appl. Phys.* **87**, 317 (2000).
2. Fu, C. *et al.* Realizing high figure of merit in heavy-band p-type half-Heusler thermoelectric materials. *Nat. Commun.* **6**, 8144 (2015).
